# Supplementary material for: Gene Expression Changes Induced by Exposure of RAW 264.7 Macrophages to Particulate Matter of Air Pollution: The Role of Endotoxins
Source: Biomolecules. 2022 Aug 10;12(8):1100. doi: 10.3390/biom12081100 (PMC9405577; doi:10.3390/biom12081100)
Supplement: Supplementary file 1 [file biomolecules-12-01100-s001.zip › biomolecules-1831193-supplementary/Roman et al_ final Supplementary Figures S1 and S2.pdf]

Supplementary Figures

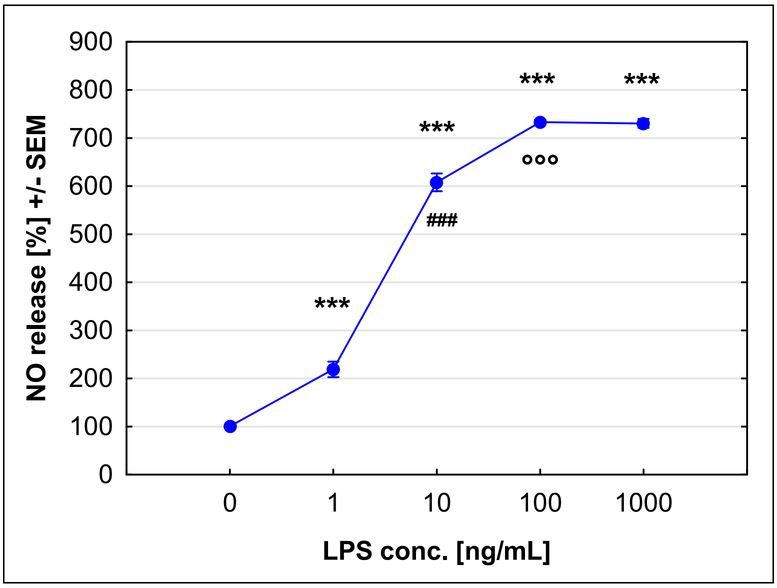

Supplementary Figure S1. Dependence of the concentration of lipopolyaccharide (LPS) in the culture medium on nitric oxide (NO) release by RAW 264.7 cells assessed with the Griess reaction after 48 hrs of culture and expressed as the percentage of the values observed in control cultures not exposed to LPS. *** $p < 0.001$ relative to control cultures, ### $p < 0.001$ vs conc. of LPS 1 ng/mL and °°° $p < 0.001$ vs conc. of LPS 10 ng/mL.

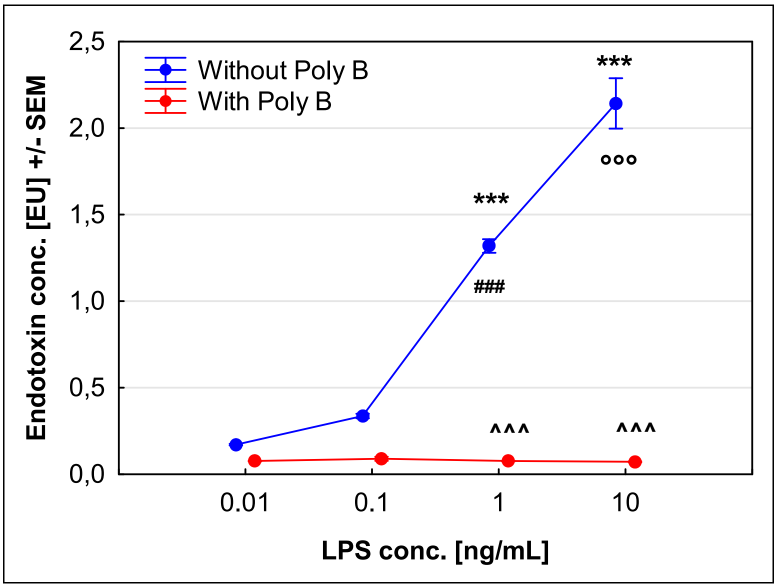

Supplementary Figure S2. The endotoxin content [EU] assessed with the Limulus Amebocyte Lysate (LAL) method in relation to LPS concentration [ng/mL] and neutralization of LPS by Polymyxin B (Poly B) at a concentration of 100 µM. *** $p < 0.001$ relative to conc. of LPS 0.01, ### $p < 0.001$ vs conc. of LPS 0.1 ng/mL, °°° $p < 0.001$ vs conc. of LPS 1 ng/mL and ^^^ $p < 0.001$ vs wells without Poly B at the same concentration of LPS.
