# Supplementary material for: Gene Expression Changes Induced by Exposure of RAW 264.7 Macrophages to Particulate Matter of Air Pollution: The Role of Endotoxins
Source: Biomolecules. 2022 Aug 10;12(8):1100. doi: 10.3390/biom12081100 (PMC9405577; doi:10.3390/biom12081100)
Supplement: Supplementary file 1 [file biomolecules-12-01100-s001.zip › biomolecules-1831193-supplementary/Roman et al_Legends to Supplementary Tables.pdf]

Legends to Supplementary Tables:

Supplementary Table S1: A table listing the results from the gene expression profiling for the PM (NIST1648a and Lap120a) and LPS treatment in RAW 264.7 macrophages. The mRNA abundance levels of 522 transcripts were found to be significantly different between the groups. For each transcript's *p*-values for time, treatment factors, all the interactions, and folds of difference between the experimental groups are presented.;

Supplementary Table S2: The results of the functional enrichment analyses performed with the Enrichr tool for the seven clusters (1–7) of genes significantly altered by PMs and LPS in RAW 264.7 macrophages (the clusters are presented in Figure 8). The table consists of enriched terms (based on KEGG 2021 Human dataset), the number of input genes in the pathway (overlap), *p* value, adjusted *p* value, odds ratio, combined score (computed as a logarithm from *p* value from the Fisher's exact test multiplied by the z score of the deviation from the expected rank), and the names of overrepresented genes. The terms with adjusted $p < 0.1$ were considered enriched and were marked in green.
